# Preparing competent graduates for delivering pharmaceutical care: an experience from Northern Cyprus

**DOI:** 10.1186/s12909-019-1875-5

**Published:** 2019-11-29

**Authors:** Abdikarim Mohamed Abdi, Arijana Meštrović, Rumeysa Demirdamar, Bilgen Basgut

**Affiliations:** 1 Department of Clinical Pharmacy, Near East University, Nicosia, Northern Cyprus, Mersin 10 Turkey; 2 Pharmaexpert Consultancy and Education, Zagreb, Croatia; 3 Faculty of Pharmacy, European University of Lefke, Nicosia, Northern Cyprus, Mersin 10 Turkey

## Abstract

**Background:**

This paper describes the implementation and evaluation of a clinical pharmacy practice (CPP) course in Northern Cyprus. The course covered a range of subjects, including internal medicine, cardiovascular and respiratory diseases, and drug information services.

**Methods:**

An 8-week structured CPP course was designed for fifth-year students. Students’ competencies were assessed using an objective structural clinical examination (OSCE) before and after the intervention. The course addressed all CPP competence domains and learning outcomes, and it utilized a wide variety of learning activities. Student perceptions, experience and preceptor evaluations were assessed using surveys.

**Results:**

Students reported that the learning objectives of the course were met. Substantial knowledge and skills in different areas of CPP were gained. A significant overall enhancement in the average grades on the OSCE was identified (23.09 ± 0.75 and 27.51 ± 0.71 out of 40). Students received the highest scores in *drug information data retrieval and interpretation* (4.4 ± 0.13), *communication skills* (4.2 ± 0.09) and *public health promotion* (3.92 ± 0.12). The lowest scores were recorded in clinical prescription management problems (2.5 ± 0.23) and *pharmacotherapy application* (2.54 ± 0.18).

**Conclusion:**

Students’ scores significantly improved from the baseline in the core competence domains. Most students found the structure, process and outcomes of the course to be beneficial and satisfactory.

## Background

Pharmacy education has experienced an extensive transformation over the last century on a global level. Dramatic changes in healthcare systems and patient needs and an evolving patient-oriented pharmacy practice have been observed [1]. The International Pharmaceutical Federation (FIP) has recently released its global vision for education and workforce development [1]. The FIP recommends that undergraduate programs equip graduate pharmacists with the adequate knowledge, skills and attitudes necessary to provide health promotion and pharmaceutical care in a variety of settings. Programs must prepare pharmacy graduates for developing the foundations of clinical knowledge and effective communication skills needed to serve individual patients [2].

In Turkey, the Higher Education Institution extended pharmacy education from a 4-year program to a 5-year program in 2005 in an effort to increase the number of courses and experiences that could contribute to students’ competencies in providing pharmaceutical care [2, 3]. The final added year is reserved for various elective courses, a graduation project, and a minimum 6-month mandatory traineeship. The traineeship is often performed under the supervision of a pharmacist in a public pharmacy or hospital [4]. Recently, after the completion of the 5-year program, a compulsory one-year preregistration training in a community pharmacy was added to the requirements, without assessing how the extra one-year educational training would strengthen the existing five-year program. In the UK and Australia, for example, students complete a 4-year undergraduate degree in pharmacy followed by a 12-month structured internship program prior to a licensing exam [5, 6].

However, introducing the concept of pharmaceutical care, student mentorship and the principles of pharmacotherapy can be challenging. The current adapted curriculum education in Turkey does not have a sufficient momentum to advance clinical pharmacy training and practice [7], since students are scarcely introduced to advanced knowledge on the nature of pharmaceutical care provision in the classroom. Topics such as disease state management, identification and resolution of drug-related problems and drug information utilization are only covered at basic levels [8]. On the other hand, pharmacists who did not receive enough clinical knowledge and training before the new regulations and reforms were implemented are now mentoring new graduates [8]. Students are conducting their practice in community pharmacies where pharmacists are often struggling with the knowledge and necessary skills required to implement patient care services [8].

Given this situation, there is a need for faculties to establish their own advanced pharmacy practice sites to enhance students’ patient care competencies to meet the increasingly complex health-care needs. This concept already exists in many countries worldwide [9].

There are over 40 pharmacy faculties in Turkey and Northern Cyprus, with local accreditations awarded by the Turkish Higher Education Counsel for the professional 5-year programs [10]. Of these, Near East University is certified by the Accreditation Council of Pharmacy Education (ACPE) [11]. To acquire this certification, the Faculty of Pharmacy reviewed its experiential programs to meet the required standards.

Advanced pharmacy practice experiences (APPEs) are practical training courses or experiences delivered in the final academic year, which enable students to strengthen their clinical skills and prepare them to take on responsibility as competent pharmaceutical care providers. Internationally, expected learning outcomes of the APPE courses are diverse and vary in length of training time and the type of settings in which the programs are taught (for example, community health centers vs. tertiary hospitals vs. community pharmacies, to mention a few) [9]. Experiential programs should be directed by qualified professionals with both academic and practice backgrounds to assure a diversity of approaches for diverse patient groups. They should include activities that foster the development of pharmaceutical care competencies rather than simply prescription dispensing. The training objectives must be based on competencies, abilities and needs. These objectives should be quantifiable for both formative and summative assessment purposes [12].

In this paper, we highlight the possible features of an experiential program that could offer additional improvements in experiential education and the competencies of pharmacy graduates to deliver effective and responsible patient care.

## Methods

### Setting and practice site

**Fig. 1:**
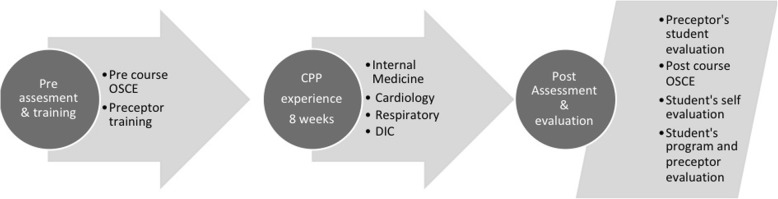
Study design and flow

In Northern Cyprus, a CPP course was established at the Near East University Hospital (NEUH) during the 2015–2016 academic year. NEUH is a tertiary university hospital that provides acute, intermediate, rehabilitation and outpatient health services. It is one of the largest healthcare centers in Northern Cyprus with 500 beds. It is also one of the leading medical facilities affiliated with the Near East University. Clinical pharmacy services were first established in the respiratory disease unit. Later, the services were extended to other clinics in cardiology, internal medicine, gynecology, geriatrics and infectious diseases. Pharmaceutical care services are provided to patients from all these clinics by the Clinical Pharmacy and Drug Information Center of the Hospital. Preceptors received training in mentoring internship students, assessing clinical competencies and applying active teaching skills. Preceptors were also required to deliver advanced ward-based pharmaceutical care services. They were required to document the outcomes over a period of 60 days before they started training 5th-year graduate students to ensure that their pharmaceutical care competencies were developed and up-to-date (Fig. 1).

### Experiential program structure

An 8-week structured clinical pharmacy practice course was designed for 5th-year students. Students received training in four modules: drug information, internal medicine, cardiology and respiratory diseases. The Center for the Advancement of Pharmacy (CAPE) within the American Association of Colleges of Pharmacy has developed CAPE outcomes as an ability-based framework or competencies to guide pharmacy educators and preceptors in setting pharmacy curricula for both didactic and practical courses’ objectives and outcomes [13]. Objectives matching the following CAPE outcomes were assigned to the course: to become a Learner, Caregiver, Manager, Promoter, Educator, Communicator, Self-aware and Professional [14]. The courses utilized a wide variety of teaching and learning activities with a minimum requirement from the students per week, including one formal and two informal case presentations, four inpatient assessments and follow ups, in-service (i.e., prepare a poster or deliver a presentation topic to the healthcare team) therapeutic newsletter preparation, two discharge patient counselling quizzes and presenting at least a journal club. Competencies targeted for strengthening included responding to symptoms and history taking, clinical prescription management problems, patient counselling skills, pharmacotherapeutic knowledge application, systems based client assessment, drug information data retrieval and interpretation, communication skills and attitudes, and promotion of public health.

### Program outcomes assessment

Student competencies in the eight main domains of the course learning outcomes were evaluated before and after the program via a formative blueprint-guided, 13-stationed OSCE. Each student was randomly assigned to one of two sets of 7 stations, one in the morning and one in the afternoon. Measures to ensure the validity and reliability of the OSCEs involved group development and a review of the case scenarios and scoring rubrics, training sessions involving the calibration of student scoring on the OSCE, and pilot testing of the OSCE stations immediately before the exam [15]. Tables 1 and 2 show the set of tasks carried out by the graduate students in the pre- and post-OSCE exams. Preceptors evaluated students’ competencies throughout the program based on daily performance, presentations, services, daily interventions, quizzes, final summative exams and a questionnaire focused on academic and clinical achievements, according to the set course objectives.

**Table 1:** OSCE station tasks in the pre-assessment

Station	Description of Task
1	Clinical prescription management in pregnancy
2	Systematic approach to patient medication history and symptoms of drug toxicity in pregnancy
3	Inspecting an adverse reaction to antihypertensive medication
4	CVD risk assessment and providing medical information
5	Systematic approach to patient medication history and symptoms for a pediatric patient with URTI
6	Compliance to an MDI drug regimen for a pediatric asthmatic patient
7	Pain assessment and management in geriatric patients
8	Clinical prescription management in a patient on levothyroxine with multiple chronic diseases
9	Inspecting DRP in a pregnant woman on antihypertensive medications
10	Educating a hypertensive patient on misconceptions about his medication
11	Counselling an asthmatic patient on PDI inhalation techniques
12	Managing the drug related problems of a sinusitis patient on decongestants who developed rhinitis medicamentosa.

**Table 2:** OSCE station tasks in the post-assessment

Station	Description of Task
1	Clinical prescription management for a patient with multiple chronic diseases and manipulation of drug information requests.
2	CVD risk assessment and medical information provision
3	Inspecting adverse reaction to a antihypertensive medication
4	Systematic approach to patient medication history and symptoms of anticoagulant drug toxicity
5	Counselling a COPD patient on hand ihaler inhalation techniques and general health measures
6	Counselling on insulin regimen for a type 1 DM patient and patient education on DM
7	Clinical prescription management for a patient on levothyroxine with multiple chronic diseases
8	Education of a T2DM patient and assessment for therapeutic goals and outcomes
9	Counselling an asthmatic patient on dry powder inhaler (PDI) inhalation techniques
10	Systematic approach to patient medication history and symptoms for a pediatric patient with upper respiratory tract infections (URTI)
11	Inspecting DRP in a geriatric patient with isosorbide dinitrate ISDN prescription and multiple morbidities with polypharmacy
12	Optimizing therapy for a T2DM patient and managing complications

### Data extraction and statistical analysis

Students evaluated program settings, sites and preceptors’ performance and their learning experience using an assessment method that incorporated a 5-domain Likert survey of 65 items. Of these, 42 items assessed the course content and perceived learned skills and attitudes. The items were summed and grouped under course objectives by 3 course educators (a professor and 2 preceptors). Students’ self-evaluation of their post course experience, the preceptors’ evaluation and grading of the students’ knowledge, skills and attitude, and the students’ pre- and post-OSCE scores were all compared and contrasted. Outcomes were analyzed using GraphPad Prism (version 6.0). The methods used to analyze the data included descriptive statistics for categorical variables. Continuous variables of the students’ scores were expressed as the mean values, mean ± standard error of the mean (S.E.M.) and were analyzed for normality using Kolmogorov-Smirnov normality tests, which showed that the pre post scores were not normally distributed. Thus, the Wilcoxon signed rank sum test was used to evaluate the pre post OSCE scores. Additionally, an unpaired t-test was utilized to compare the preceptors’ evaluation of students and students’ self-reported evaluation of their achievement of course objectives. The level of significance was set at p < 0.05.

## Results

**Fig. 2:**
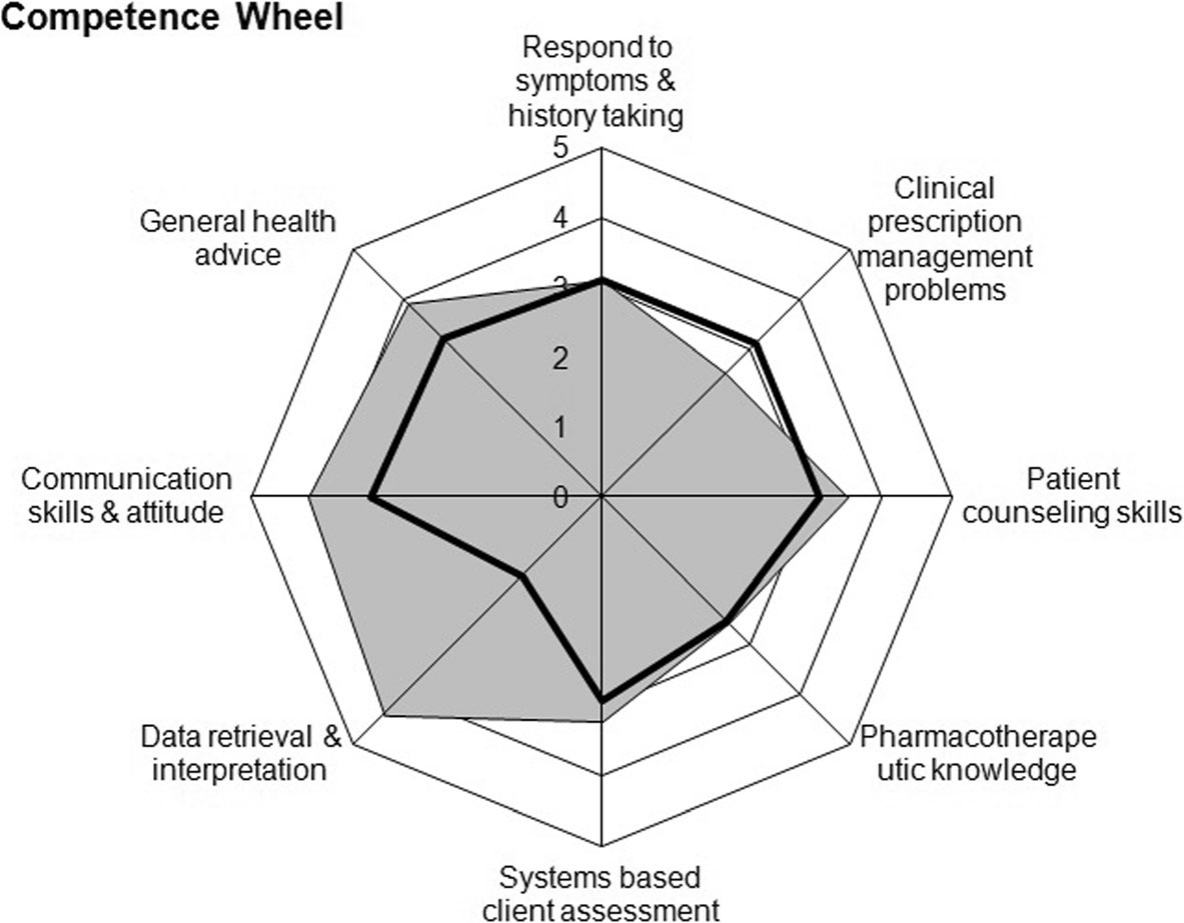
Students’ performance mapped on a competence wheel, compared to the baseline

A total of 81 students completed the Clinical Pharmacy Practice Experience program. Of these students, only 74 students attended both pre- and post-OSCE examinations and were evaluated by the analysis. All 81 students attended the final summative exam and were assessed individually by the preceptors. The lowest and highest grades achieved on the baseline OSCE evaluation were 3/40 and 37/40, respectively, while 10/40 and 39/40, respectively, were the scores on the final assessment. The average rounded grades in the first and final assessments were 23.09 ± 0.75 and 27.51 ± 0.71 (out of 40), respectively, demonstrating an overall enhancement (p < 0.001) (Fig. 2). Within each station, several skills and attitudes were assessed for each case. In the final assessment of post-practice experience, the average improvement in scores was significant in the *drug information data retrieval and interpretation* (P < 0.001), *communication skills* (P < 0.001) and *promoting public health* (P = 0.02) when compared to the baseline OSCE scores. Additionally, the *pharmacotherapy knowledge application* and *patient counseling* skills scores improved, but not significantly compared to the initial assessment. The students’ scores on both OSCEs were not significantly affected by the set of seven stations to which they were randomly assigned. Table 3 shows the average scores for the course’s eight main competencies, as assessed by the OSCE stations.

**Table 3:** Students’ average performance on the OSCE’s assessed competences post-APPE

Competences	Mean Score out of 5 ± SEM Pre- APPE	Mean Score out of 5 ± SEM Post- APPE	*p*-value*
Respond to symptoms and history taking	3.1 **±** 0.2	3.1 ± 0.2	*p* = 1.000
Clinical prescription management problems	3.1 **±** 0.25	2.5 ± 0.23	*p* = 0.220
Patient counselling skills	3.1 **±** 0.14	3.5 ± 0.13	*p* = 0.165
Pharmacotherapeutic knowledge application	2.5 **±** 0.12	2.54 ± 0.18	*p* = 0.567
Systems-based client assessment	2.9 **±** 0.3	3.2 ± 0.25	*p* = 0.577
Data retrieval and interpretation	1.6 **±** 0.12	4.4 ± 0.13	*p* < 0.001
Communication skills and attitude	3.3 ± 0.15	4.2 ± 0.09	*p* < 0.001
Promoting public health	3.2 ± 0.18	3.92 ± 0.12	*p* = 0.02
Average	2.85 ± 0.18	3.42 ± 0.17	*p* < 0.001

* Evaluated using the Wilcoxon signed rank sum test

### Students and preceptor evaluations

A total of 36 students completed the student perception survey (44%). Students reported that the following objectives were met the most during their experiential practice: “*Searching for drug information in a timely manner by using evidence-based resources*”, *“Being capable of providing public heath advice and creating awareness of general health issues”* and *“Patient interviewing, education and counseling”* (3.37 ± 0.17; 3.29 ± 0.15; 3.21 ± 0.15, out of 5 respectively). However, *“To be knowledgeable in drug therapy planning and evaluation in hospitals”* was identified as less enhanced compared to the other objectives (3.09 ± 0.18 out of 5). Table 4 shows students’ self-evaluation and the preceptor evaluation of students’ performance based on course objectives and matching CAPE outcomes.

**Table 4:** CAPE 2013 outcomes matched with course objectives

CAPE 2013 outcomes	Course objectives and outcomes	Preceptor evaluation	Student self-evaluations	*p*-value*
1.1 Learner	“To be capable of disease state management and monitoring for therapeutic endpoints in different clinical settings”	3.11 ± 0.10	3.12 ± 0.16	*p* = 0.3237
2.1 Caregiver	“To be knowledgeable in drug therapy planning and evaluation in hospitals”	2.90 ± 0.1	3.09 ± 0.18	*p* = 0.9567
2.2 Manager	“Searching for drug information in a timely manner by using evidence-based resources. Critique primary, secondary and tertiary resources.”	3.25 ± 0.10	3.37 ± 0.17	*p* = 0.2852
2.3 Promoter	“Be capable of providing public heath advice and creating awareness of general health issues.”	3.47 ± 0.09	3.29 ± 0.15	*p* = 0.5237
3.2 Educator	“Patient interviews, education and counseling.”	3.10 ± 0.10	3.21 ± 0.15	*p* = 0.5420
3.6 Communicator	“Effectively communicate verbally and nonverbally in patient cases and drug information presentations and in communication with other healthcare providers.”	3.37 ± 0.09	3.19 ± 0.18	*p* = 0.3202
4.1–4.4 Self-awareness and Professionalism.	“Professionalism in all aspects of practice, including team interaction, motivation, communication skills, reporting and service documentation.”	3.23 ± 0.10	3.19 ± 0.17	*p* = 0.8315

* Evaluated using an unpaired t-test

Most of the students perceived the preceptors’ individual student evaluations as affirmative and the preceptors as knowledgeable in their practice area (60 and 54%, compared with 14.30 and 22.9% who disagreed, respectively). Furthermore, the students also perceived the university hospital as a suitable practice site that fosters their learning and practice (52% agreed, while 26% disagreed). Informal case presentations were identified as the learning activity that was the most beneficial (27%), although all learning activities appeared to be beneficial for some students (36%) (see Additional file 1: Table S1). Most of the students preferred multiple choice questions in the final summative exams than other forms of assessment (50%). The majority of students rated the clinical pharmacy practice experience introduced in this study as the practice course with the highest impact on their experience from all the experiential courses (67%, Additional file 2: Table S2).

## Discussion

Globally, pharmacy practitioners are trained to provide a wide range of pharmaceutical care services as well as to promote wellness and public health [1]. This can be achieved by adapting educational programs that involve sufficient practice experiences in different settings, giving the students the opportunity to practice and refine the skills they have learned in the classroom [12]. Pharmacy practice experiences constitute the work-based learning experience in pharmacy education, which provides students with the opportunity to graduate from pharmacy school with the ability to meet the changing needs of the profession in different settings and the requirements of those who are served by the profession [9].

The 8-week structured clinical pharmacy practice course was designed for 5th-year students. The course consisted of eight stated course objectives and outcomes that fall within the key CAPE 2013 recommended outcomes, as shown in Table 4. After the APPE course, students were generally observed to become more competent in conducting activities related to the domains of being Learners, Caregivers, Educators and Self-awareness but more significantly in conducting activities related to being Communicators, Problem solvers, Promoters and Professionals. To assess such competences (i.e., skills, knowledge, values and attitudes), Bloom’s taxonomy categorizes cognitive skills as knowledge, comprehension, application, analysis, synthesis, and evaluation. Thus, experiential practices are assumed to be categorized at the top of the taxonomy, whereby preceptors should evaluate students on their ability to synthesize and evaluate information to optimize the therapy outcomes for their patients [16].

Different methods are used worldwide to evaluate experiential pharmacy practice courses. Some courses use a combination of evaluation methods, including arbitrary assignments of grades and written, verbal or practical examinations, and observation ratings and graded assignments [17]. In Turkey and Northern Cyprus, students are mainly graded on a final verbal exam, with or without student portfolio files. Donald Kirkpatrick developed a four-level training evaluation model (reaction, learning, behavior and results) to evaluate the overall effectiveness of training programs that are also applicable to experiential education. A recently published review evaluated the APPE in the United States using Kirkpatrick’s hierarchy (KH) model. The authors reported that more than two-thirds of the programs are assessed using lower levels of KH [18]. Table 5 shows the distribution of activities conducted in the assessment of the APPE in this study.

**Table 5:** Distribution of activities conducted in the assessment of the APPE

Levels of assessment using Kirkpatrick’s hierarchy (KH)	Level 1. Reaction	Level 2. Learning	Level 3. Behavior	Level 4. Results
All assessment activities	• Student self-enhancement survey • Preceptor student learning outcomes evaluation survey	• Quizzes • Written final exam	• Case discussions • Informal and formal case presentations • Simulation and actual patient counseling • Journal clubs • OSCE’s	• Clinical interventions • In-services
Course activities’ scores weight %	0	40%	40%	20%

The program was evaluated using multiple levels of evaluation based on the KH during the course. Students in the current study regarded all the different assessment methods used as beneficial for their learning (36%), while case discussions were indicated as being of the most benefit to other students (27.3%). The students objectively evaluated the beneficial impact of experiential practices, indicating that this program was the most beneficial among the courses they took (65.7%).

Students in the OSCE have shown significant overall enhancement in post experience competences, such as *communication skills, data retrieval and interpretation, and public health promotion*. Improvements in drug information data retrieval and interpretation skills were attributed to the skills and practical experiences gained through the newly established Drug Information Center at the University Hospital [19].

According to the students’ evaluations, preceptors had good background knowledge and evaluated students individually in a satisfactory manner (60 and 54.7%, respectively), while one quarter of the surveyed students (25.7%) expressed doubts about the preceptors’ interest in teaching, as well as about the access to the necessary patient information in the practice institution. As the role of the preceptors is crucial for the success of an APPE, preceptor training and encouragement by faculty administrations can contribute to preceptors’ proactiveness and performance. Moreover, students’ own self-assessment reports resemble their actual external evaluation scores, showing the effectiveness of the APPE course. Multiple assessment methods were used for evaluating the outcomes of the program, including OSCEs. Dennis et al. (2016) reported that out of 91 articles published in Medline on APPE introduction and evaluation; approximately 60% used only qualitative assessments such as students’ self-evaluations and perceptions or preceptor evaluations [17]. Sturpe mentioned that only 37% of the sampled pharmacy schools in the United States were using OSCEs in their curricula in 2009 [20].

Potential benefits of objective evaluations were reported regarding student knowledge pre- and post-APPEs using formative assessments. In addition to providing preceptors with the chance to individualize the student experience, pre post assessments can provide further evaluations of students’ learning achievement during APPEs. Pre post assessments are globally regarded as a gold standard in student academic and clinical evaluations because they enable preceptors, educators and researchers to examine the evidence on academic and clinical progress from the previous levels, for example, to increase the quality of APPEs [21]. Masters et al. (2012) observed a significant mean student improvement of 23.6% from the pretest, while Harris et al. (2016) reported a 21.2% improvement in scores from a written pre post test for students of ambulatory APPE, while a relatively lower mean improvement was observed in our study’s student scores (11%) on the pre post OSCE evaluation [22, 23].

The OSCEs revealed an overall enhancement in students’ overall performance, with a significant enhancement of *communication skills, data retrieval and interpretation* and *public health promotion*. However, the skills were only partially enhanced in *patient counseling and pharmacotherapy knowledge application* and no enhancement but a partial worsening was found in *clinical prescription management problems*. Because identifying and resolving drug-related problems is closely related to *pharmacotherapy knowledge application,* more learning activities to strengthen both of these skills are recommended as well as a longer practice for the development of such competences.

### Limitations

Several limitations of this study are important to mention. The low response rate for the self-assessment survey was mainly attributed to the length of the survey (65 items), although a response rate of 20% from non representative samples is considered acceptable for generating further hypotheses and recommendations [24]. Additionally, the lack of critical evaluation of the students’ impact on the healthcare setting is a limitation for this study, although positive benefits of APPE courses for improving the quality of care for patients have been observed elsewhere [25]. Apart from the calibration of the students’ scores on the OSCE exams carried out in this study, other forms of assessment were not calibrated during the preceptor training session, and this could have undermined the consistency of the students’ assessment during this course.

Nonetheless, this is the first study reporting the experience of a structured experiential practice for pharmacy graduate students from Northern Cyprus and Turkey. Both summative and formative assessment methods were adopted, and both objective and subjective approaches were used to evaluate student performance and all components of the program. Sufficient measures were carried out to ensure the reliability and consistency of the OSCE exams. Further studies should assess the barriers to successful APPEs in this region. Such studies should develop experiential practices in multiple diverse settings to optimize the care provided by new pharmacy graduates in Turkey and Northern Cyprus.

## Conclusion

The course provided a rich experiential learning and teaching environment for pharmacy graduate students. We used the global literature on pharmaceutical care practices to inform the program’s curriculum activities. The competences of the students were strengthened in the domains of *drug information data retrieval and interpretation*, *communication skills* and *promoting public health*. However, no significant improvement was observed in the *pharmacotherapy knowledge application* and *clinical prescription management problems* scores compared to the initial assessment, which necessitates more activities and a longer period of time for the development of these competences and highlights the need for further training and course development. Overall, most of the surveyed students perceived the structure, process and outcomes of the course as beneficial and satisfactory. These outcomes should be researched further by larger and more representative studies.

## Supplementary information

**Additional file 1: Table S1.** Students site and preceptor evaluation

**Additional file 2: Table S2.** Student’s perception of assignment, assessments and experiential practices

## Data Availability

The data sets supporting the conclusions of this article are available in excel file and can be provided if requested.

## Abbreviations

ACPE: Accreditation Council of Pharmacy Education
APPE: Advanced Pharmacy Practice Experience
CAPE: Initiative of the Center for the Advancement of Pharmacy
CPP: Clinical Pharmacy Practice
CVD: Cardiovascular Diseases
DI: Drug Information
DRP: Drug related problems
FIP: International Pharmaceutical Federation
KH: Kirkpatrick’s learning evaluation hierarchy
MDI: Metered Dose Inhaler
OSCE: Objective Structured Clinical Examination
PDI: Powdered Dose Inhaler
S.E.M: Standard Error of the Mean
UK: United Kingdom
URTI: Upper Respiratory Tract Infection
USA: United States of America
