# Supplementary material for: Preparing competent graduates for delivering pharmaceutical care: an experience from Northern Cyprus
Source: BMC Med Educ. 2019 Nov 29;19:442. doi: 10.1186/s12909-019-1875-5 (PMC6883527; doi:10.1186/s12909-019-1875-5)
Supplement: Supplementary file 1 — Additional file 1: Table S1. Students site and preceptor evaluation [file 12909_2019_1875_MOESM1_ESM.docx]

**Additional file 1: Students site and preceptor evaluation**

|  | **Preceptors and site** | **disagree** | **Neutral** | **Agree** |
| --- | --- | --- | --- | --- |
|  | The preceptor was interested in teaching this practice. | 25.70 | 25.7 | 48.6 |
|  | The Amount of information about the practice given for the students was sufficient | 22.90 | 34.3 | 42.9 |
|  | The goals and objectives of the practice were outlined and/or explained at the beginning of the practice. | 17.10 | 34.3 | 48.6 |
|  | I had adequate patient and preceptor contact on this practice to meet the learning objectives. | 14.30 | 40.0 | 45.7 |
|  | I had access to necessary patient information and was involved in managing cases and resolving therapeutic problems. | 25.70 | 28.6 | 45.7 |
|  | The preceptor was readily available to answer questions and concerns. | 22.80 | 28.6 | 48.7 |
|  | Good direction and feedback were provided. | 22.80 | 25.7 | 48.6 |
|  | The preceptor was knowledgeable in their response to questions and approach to therapy. | 22.90 | 22.9 | 54.4 |
|  | This preceptor evaluated me at the end of the rotation in a manner which was helpful to me | 14.30 | 25.7 | 60 |
|  | This preceptor served as a role model for a pharmacist practicing in this practice setting. | 25.70 | 25.7 | 48.6 |
|  | The NEU hospital practice provided an environment (physical and philosophical) that facilitated my learning and valuable for students | 25.70 | 22.9 | 51.5 |
