# Supplementary material for: Preparing competent graduates for delivering pharmaceutical care: an experience from Northern Cyprus
Source: BMC Med Educ. 2019 Nov 29;19:442. doi: 10.1186/s12909-019-1875-5 (PMC6883527; doi:10.1186/s12909-019-1875-5)
Supplement: Supplementary file 2 — Additional file 2: Table S2. Student’s perception of assignment, assessments and experiential practices [file 12909_2019_1875_MOESM2_ESM.docx]

**Additional file 2: Student’s perception of assignment, assessments and experiential practices**

| Statements | | Disagree | | Neutral | Agree | |
| --- | --- | --- | --- | --- | --- | --- |
|  | The students’ assignment procedure and placing was fare and uncomplicated | 27.2 | | 30.4 | 42.4 | |
|  | Which evaluation approach was most beneficial for you | quiz | Formal Case presen-tations | Final exam | Informal case discussion | All |
|  |  | 6.1 | 15.2 | 15.2 | 27.3 | 36.3 |
|  | The final exam questions were suitable for the assessment of knowledge and skills obtained from this practice. | 20.6 | | 31.4 | 47.0 | |
|  | The final exam questions were clear challenging but not too much difficult | 35 | | 24.2 | 45.4 | |
|  | Best assessment method as a final exam | all | Assay | OSCE | Oral examination | Multiple choices |
|  |  | 8.8 | 5.9 | 17.6 | 17.6 | 50 |
|  | **General perceptions about experiential practices** | | | | | |
|  | Pharmacy practice experiences were essential and important for being professional pharmacist. | 25.7 | | 25.7 | 48.5 | |
|  | **Your evaluation to pharmacy practice courses positive impact on your practice development** | | | | | |
|  | Pharmacy practice NEPHAR 490 | 15.6 | | 31.3 | 53.1 | |
|  | Pharmacy practice NEPHAR 491 | 22.9 | | 28.6 | 48.6 | |
|  | Pharmacy service clinical rotations NEPHAR 802 | 17.1 | | 17.1 | 65.7 | |
|  | Pharmacy practice NEPHAR 590 | 25.8 | | 20.0 | 54.3 | |
|  | Experiential practices provide students with essential knowledge skills and attitude to take the role of pharmacist professionally | 23.5 | | 17.6 | 58.8 | |
